# Point-of-care Diagnostic Framework for Fibromyalgia Using Integrated Vibrational Spectroscopy and Metabolomics

**DOI:** 10.21203/rs.3.rs-9873664/v1

**Published:** 2026-06-11

**Authors:** Shreya Madhav Nuguri, Luis Rodriguez-Saona, Chengyu Gao, Haona Bao, Katherine R. Sebastian, Michelle M. Osuna-Diaz, Monica M Giusti, Lianbo Yu, Silvia De Lamo Castellvi, Kevin V. Hackshaw

**Affiliations:** 1 Department of Internal Medicine, Dell Medical School, The University of Texas, 1601 Trinity St, Austin, TX 78712, USA; 2 Department of Food Science and Technology, The Ohio State University, Columbus, OH 43210, USA; 3 Mass Spectrometry and Proteomics Facility, The Ohio State University, Columbus, OH, USA; 4 Campus Sescelades, Departament d’Enginyeria Química, Universitat Rovira i Virgili, Av. Països Catalans 26, 43007 Tarragona, Spain; 5 Center of Biostatistics and Bioinformatics, The Ohio State University, Columbus, OH 43210, USA

**Keywords:** Vibrational spectroscopy, Metabolomics, Translational, Point-of-care diagnosis, Fibromyalgia

## Abstract

**Background:**

There is a critical need for objective diagnostic strategies for syndromes that rely on subjective questionnaires to ensure accurate and reliable diagnosis. Fibromyalgia (FM), one of the most common rheumatic disorders, remains particularly challenging to diagnose because of symptom overlap with related conditions, especially rheumatoid arthritis (RA). This exploratory study evaluated the feasibility of a combined spectroscopic–metabolomic workflow for distinguishing FM from RA and healthy controls (HC).

**Methods:**

Whole blood specimens were analyzed from 40 patients with FM, 20 patients with RA, and 10 HC participants. The analytical workflow combined portable Fourier-transform infrared (FTIR) spectroscopic fingerprinting with mass spectrometry (MS)-based metabolite identification. Potential confounding factors affecting analytical signatures were systematically evaluated, and alternative extraction protocols were compared. Methanol (MeOH) and methanol/1-butanol (MeOH/BuOH) extraction methods gave a good metabolome coverage and were selected for subsequent analyses. Soft independent modeling by class analogy (SIMCA) and partial least squares discriminant analysis (PLS-DA) were used for classification, while partial least squares regression (PLSR) was used to correlate FTIR spectral features with biologically relevant metabolites identified by MS.

**Results:**

SIMCA models demonstrated good classification between FM and HC, with interclass distances (ICD) exceeding 4.1 for MeOH extraction and 4.6 for MeOH/BuOH extraction. MS-based metabolomic analyses identified oligopeptides, inosine monophosphate, and signaling lipid molecules as major contributors to group differentiation, suggesting probable dysregulation of oxidative stress and inflammatory signaling pathways, as well as purine, amino acid, and free fatty acid metabolism. PLSR models showed strong correlations between FTIR spectral data and MS intensities, including inosine monophosphate, N-acylethanolamines (NAE), monoacylglycerols (MAG), N-fructosyl phenylalanine, N-fructosyl isoleucine, Ser-Phe, and N-acetylhistidylprolinamide (R ≥ 0.79; SECV ≤ 0.30).

**Conclusions:**

These findings demonstrate the potential of integrating rapid FTIR spectroscopic fingerprinting with MS-driven metabolomics to identify biologically relevant signatures associated with fibromyalgia. The strong classification performance and metabolite correlations support the potential translation of this diagnostic pipeline into a rapid, point-of-care approach for objective FM diagnosis.

## Graphical abstract

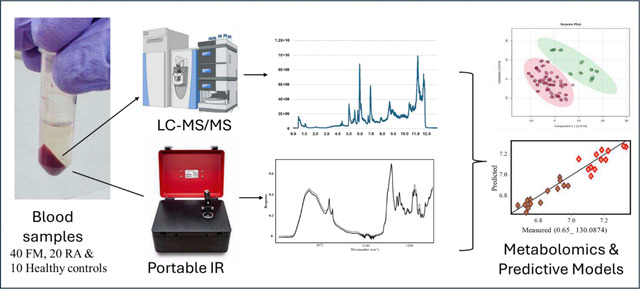

## 1. Introduction

Fibromyalgia (FM) is a chronic pain disorder marked by widespread musculoskeletal pain with associated fatigue, sleep disturbance, cognitive impairment, autonomic dysfunction, and mood symptoms, affecting approximately 2–4% of the global population [1–3]. Despite its high prevalence and substantial impact on quality of life, FM remains difficult to diagnose and manage due to its poorly understood etiopathogenesis and the absence of validated biomarkers or objective diagnostic tests [4]. Current diagnostic criteria use combined scores from the Widespread Pain Index (WPI) and the Symptom Severity (SS) scale to capture the overall clinical impact of fibromyalgia on affected individuals [5] and may take up to five years to receive a definitive diagnosis of FM [6]. Delayed diagnosis contributes to increased healthcare expenditures driven by repeated clinical consultations, costly and often uninformative diagnostic testing, and the use of minimally effective treatments [7]. The development of a reliable, objective method for earlier diagnosis could substantially improve patient outcomes, reduce healthcare costs, and enhance quality of life for individuals with FM [8–10].

Despite increasing interest in metabolomics and vibrational spectroscopy for disease diagnostics, few studies have integrated spectroscopic fingerprinting with mass-spectrometry metabolomics within a unified workflow designed for point-of-care translation. Spectroscopic techniques such as FTIR provide rapid biochemical signatures but lack direct molecular specificity, whereas LC-MS offers high molecular resolution but requires extensive laboratory infrastructure. Combining these complementary approaches may enable the development of rapid screening tools guided by molecularly validated markers.

Our group has previously demonstrated that vibrational spectroscopy can distinguish fibromyalgia from related syndromes including rheumatoid arthritis, systemic lupus erythematosus, osteoarthritis, chronic low back pain, and long COVID using chemometric analysis of blood-derived spectra [4,8–13].

In one of our previous studies, we evaluated different extraction approaches using blood aliquots sampled on dried bloodspot cards (DBS) including aqueous extract (blood), acetonitrile-based protein precipitation, and ultrafiltration [9]. We later showed that DBS process blanks exhibits significant contributions from waxy and pectic constituents of the Whatman card support, whereas the VAMS process blank exhibited a very low response signal, ensuring that that the spectral information was unique to the sample and not influenced by external artifacts [13]. Consequently, classification models were redeveloped using VAMS samples processed by ultrafiltration, the resulting classification models demonstrated the best performance in the functional group (2600–3680 cm^−1^) and fingerprint (1740–785 cm^−1^) region. The 2600–3680 cm^−1^ originates from C-H stretching vibrations typical of hydrocarbon chains [14], however, ultrafiltration suffers from a significant loss of hydrocarbon moieties when compared with solvent precipitation [15].

Solvent precipitation with organic solvents is the most used method for global metabolomics by LC-MS. Organic solvents precipitate proteins and disrupt metabolite-protein binding in blood samples, while providing excellent metabolome coverage and repeatability [15]. In this study, solvent precipitation using different organic solvents will be investigated to improve the inclusion of hydrocarbon species and enhance signals associated with C-H stretching.

Extracting metabolites from samples dried on a VAMS substrate using different solvents is a form of solid-phase extraction. The VAMS substrate possesses both ionic and hydrophobic properties, which creates non-specific binding to many analytes in the sample [16]. Accordingly, Neoteryx Mitra has provided guidelines recommending various solvents to reduce these different interactions [16,17]. In organic extraction method, when VAMS tips are exposed to a water miscible solvent, proteins in the blood precipitate on the VAMS substrate while small organic soluble components are eluted [17]. Thus, beyond being merely a sampling matrix, VAMS devices are an integral part of the sample preparation process [17].

VAMS was developed in 2014 and since then numerous studies have investigated the measurement of analytes from VAMS using various bioanalytical instruments including mass spectrometry and immunoassay [18–22]. Our research group has been pioneering the use of VAMS substrate extracts for vibrational spectroscopy analyses, including infrared and Raman spectroscopy [8,13]. However, there remains a lack of a well-defined protocol for metabolite extraction from the VAMS substrate, particularly for untargeted analysis using vibrational spectroscopy.

In this proof-of-concept study, we evaluate various solvents, as suggested by the Mitra guidelines, for chemical extraction from the VAMS substrate and evaluate their feasibility for analysis by vibrational spectroscopy. The portable characteristics of FTIR make it well suited as a point-of-care device, with minimal sample manipulation by users. Hence, FTIR was considered in this study, although the findings can be extrapolated to analysis by Surface Enhanced Raman spectroscopy (SERS). The modified protocol will be applied to whole blood samples, and the performance of whole blood extracts will be compared to that of dried blood on VAMS substrate. The protocol will be applied to a small cohort of samples to develop classification algorithms using infrared spectral data, discriminating FM from Healthy Controls (HC) and clinically similar syndrome, Rheumatoid arthritis (RA). To our knowledge, this is the first study integrating portable FTIR spectroscopy with LC-MS metabolomics within a unified workflow designed for point of care fibromyalgia diagnostics.

Thus, the objective of this research is to improve existing protocol for small molecule extraction from both VAMS samples and whole blood, to enhance spectroscopic signals while making the workflow simpler and faster, and integrate multivariate data from powerful fingerprinting techniques, MS and FTIR to develop preliminary regression algorithms for point-of-care diagnosis of FM syndrome.

We hypothesized that optimizing metabolite extraction from VAMS and whole blood samples would enhance FTIR spectral signatures and enable integration with LC-MS metabolomics to identify metabolite features underlying spectroscopic discrimination.

## 2. Results

An ideal extraction methodology for untargeted metabolomics would provide comprehensive recovery of a wide range of metabolites while remaining simple and fast to minimize metabolite loss or degradation [23]. Nonetheless, achieving this is challenging due to variations in chemical mass, physicochemical properties and the broad range of metabolite concentrations [23]. Moreover, developing a one-size-fits-all extraction procedure is difficult [17]. Extraction protocols depend on the biofluid sampled (serum, urine, plasma, whole blood or oral fluid) on the VAMS substrate, the target analyte or analyte panel (hormones, drugs, metabolites, antibodies or proteins) and the compatibility of the extraction solvent with both the analyte and the analytical instrument used [16,17]. Given the exploratory nature and limited cohort size, the models presented here are intended to evaluate analytical feasibility rather than establish clinical diagnostic performance.

### 2.1 Identifying potential interferences from sample collection tubes

**Figure 1:**
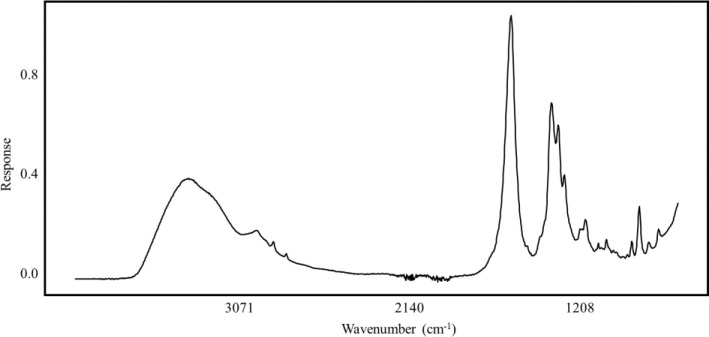
FTIR spectra of MeOH extract from a process blank (nanopure water collected in EDTA anticoagulant tube and sampled on a VAMS tip).

Purple-capped blood collection tubes coated with ethylenediaminetetraacetic acid (EDTA) anticoagulant are widely used for routine laboratory examinations [24]. EDTA prevents blood clotting by chelating ions in the blood that are essential for coagulation [24]. Figure 1 shows the MeOH extract spectra from process blank tips (nanopure water collected in EDTA anticoagulant tube and sampled on a VAMS tip). These results demonstrate that the EDTA additive is extracted from the VAMS substrate and can significantly confound FTIR signals of the sample extract. EDTA tubes are marked with a fill line to ensure the correct blood-to-anticoagulant ratio for accurate pathological testing [25]. However, improper filing, either under-filling or over-filling during phlebotomy leads to spectral inconsistency, introduces confounding interferences and results in errors during multivariate statistical analysis. The EDTA-related signals are inconsistent across samples and therefore cannot be reliably removed or subtracted during data processing. Nevertheless, the EDTA did not influence the results of the MS analysis. To ensure compatibility with both analytical techniques, blood was collected in additive-free tubes. Subsequently, various extraction solvents were evaluated to determine their compatibility for an omics-based approach.

### 2.2 Spectroscopic evaluation of extraction procedures using various solvents

To determine the most suitable extraction protocol for VAMS- based metabolomics targeting global metabolome coverage, 7 different organic solvents as discussed in Materials and methods section were selected for extraction and infrared signals from the resulting extract were compared. Mitra recommends the use of 1% FA and 1% NH_4_OH for acidic and basic analyte, respectively [16]. Extraction efficiency can be improved by adjusting the pH of the extractant using FA or NH_4_OH to neutralize charged moieties and disrupt non-specific ionic binding to the substrate, thereby facilitating analyte release from the matrix [16]. In addition, FA has been shown to disrupt noncovalent interactions involving protein-bound metabolites, further improving extraction efficiency [26]. Water has also been suggested as an extractant in certain cases, particularly when analytes are located within cells and solvent-induced protein denaturation is to be avoided [16].

**Figure 2:**
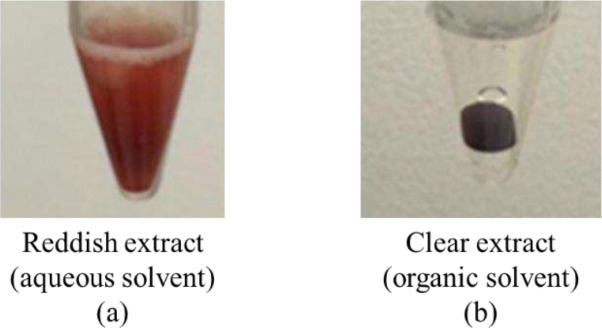
Extraction solutions of dried blood sampled on a VAMS tip using aqueous and organic solvents. The aqueous based solvent produced a red-brown extract, while the organic based solvent extracts were clear.

The addition of 1% FA or 1% NH_4_OH produced a maroon-colored extract due to hemolysis of red blood cells (RBCs), primarily caused by significant changes in pH and osmotic pressure [27]. Pure water, being hypotonic, causes complete hemolysis of blood samples through osmosis, resulting in a reddish colored extract (Figure 2a) [28].

**Figure 3:**
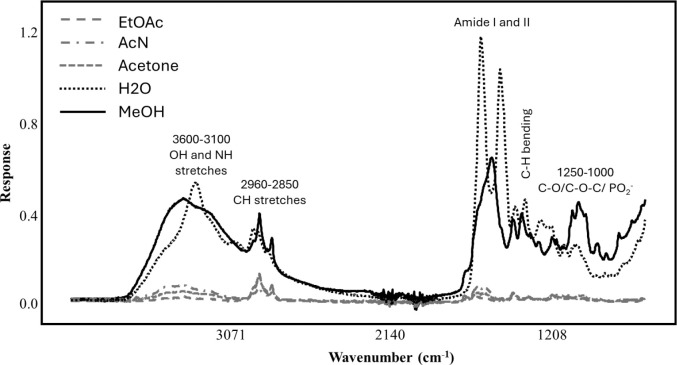
FTIR spectra of dried blood extracts obtained using water and organic solvents (EtOAc, AcN, Acetone and MeOH)

In vibrational spectroscopy analysis, inhomogeneous or particulate samples scatter or absorb light inconsistently, leading to spectral distortion and reduced reproducibility [15,29]. Furthermore, large components, such as the RBCs can dominate and mask signals from small molecules of interest, thereby reducing sensitivity and affecting infrared and Raman spectral measurements (due to fluorescence and scattering effects) [30,31]. This is evident from the spectral pattern and intensity of the water extract shown in Figure 3, where signals from unwanted cellular components (large components) dominate and obscure the spectral features of small molecules extracted using organic solvents. In addition, aqueous extracts are more complex and may interfere with the accuracy of bioanalytical assays [17]. Consequently, aqueous extracts often require further purification steps, such as solid phase extraction (SPE), liquid-liquid extraction (LLE), filtration or protein precipitation, to remove or reduce these interfering components and obtain a clearer extract [16,17]. These additional steps increase overall sample-preparation time and complexity, potentially leading to metabolite losses and selective screening of metabolites [15]. Hence, water miscible organic solvents were evaluated to precipitate proteins and obtain a clean extract.

When dried blood on VAMS tips is exposed to water miscible organic solvents, proteins precipitate and become trapped within the tip, while small molecules elute [17]. All organic solvent extracts were clean and required no further sample preparation. Acetone, acetonitrile and ethyl acetate produced lower signal intensities compared with MeOH extract, as illustrated in Figure 3. Methanol extracts exhibited rich spectral signatures and high intensity across both the regions, functional group (3700 – 2700 cm^−1^) and fingerprint region (1800 – 750 cm^−1^). Table 1 summarizes the key findings using various extractants.

A fully hemolyzed solution obtained using water as the extractant releases intracellular analytes, and subsequent [19]aqueous extraction followed by protein precipitation further disrupts analyte-protein associations, liberating them [16]. Therefore, MeOH was used to precipitate proteins from the water extract, however, the resulting solution and the dried pellet were turbid and contained hemolyzed particles (**Figure S1a**), making them inadequate for spectroscopic analysis. Biphasic methods enable the separation of aqueous metabolites from lipids using immiscible solvents, although each phase contains fewer metabolites, separate analysis followed by data integration provides greater overall metabolite coverage [32]. While the lipid fraction was clear, the aqueous fraction contained hemolyzed particles **(Figure S1b)**. Biphasic extraction using the Matyash method involves three times the volume of MTBE compared to MeOH [33]. The high concentration of the organic solvent MTBE leached additives from laboratory plasticware [34] which interfered with the IR response.

It has been reported that mixed solvent systems outperform single solvents because of broader polarity coverage [35]. Extractants with different polarity index were prepared by combining methanol with other solvents, and their effects on infrared profiles were compared. A 90% MeOH extractant is more polar than pure MeOH and is therefore expected to extract a greater proportion of polar metabolites. However, comparison of the spectra showed that the 90% MeOH extract exhibited a spectral pattern and intensity nearly identical to those of pure MeOH.

Lipophilic organic solvents are required to extract neutral lipids, therefore, MTBE, was incorporated into the extractant mixture to facilitate the extraction of hydrophobic compounds [36]. The monophasic extractant mixture containing 70:20:10 MeOH/MTBE/water exhibited slightly more defined signals in the functional group region (3000 – 2800 cm^−1^, primarily corresponding to C-H stretching) and comparable intensities in other infrared regions. The addition of 10% water likely increased the polarity of the mixture, making it closer to that of pure methanol.

Acetonitrile, a polar aprotic solvent, is less polar than MeOH which is polar protic, thus, a 70:30 MeOH/AcN is slightly less polar than pure MeOH. The mixture yielded well-defined nonpolar spectroscopic signatures (3000 – 2800 cm^−1^, C-H stretching vibrations, and 1730 cm^−1^, C=O stretching), although lower intensities were observed in other parts of the fingerprint region. Another polar aprotic solvent, EtOAc is less polar than AcN, was used to further reduce the polarity of the extractant. A 50:50 MeOH/EtOAc extractant provided enhanced definition of nonpolar spectroscopic signatures but resulted in reduced intensities in the fingerprint region.

Further reducing the polarity of the extractant using MTBE mixtures such as 70:30 MeOH/MTBE and 70:20 MeOH/MTBE produced well -defined nonpolar spectroscopic signatures (3000–2800 cm^−1^, C-H stretching vibrations and 1730 cm^−1^, C=O stretching), though lower intensities were observed in other parts of the fingerprint region. To efficiently extract both polar and non-polar metabolites from the matrix, the tip was first extracted using 90% MeOH to dissolve polar metabolites, followed by extraction using MTBE to extract non-polar molecules. Although this approach resulted in well-defined nonpolar spectroscopic signatures, it yielded lower intensities in fingerprint region. Table 2 illustrates the FTIR spectra obtained using the aforementioned solvent mixtures, compares them with the FTIR spectra of the pure MeOH extract and summarizes the key observations.

### 2.3 FTIR signal of methanol extract from dried blood on VAMS substrate and whole blood

**Figure 4:**
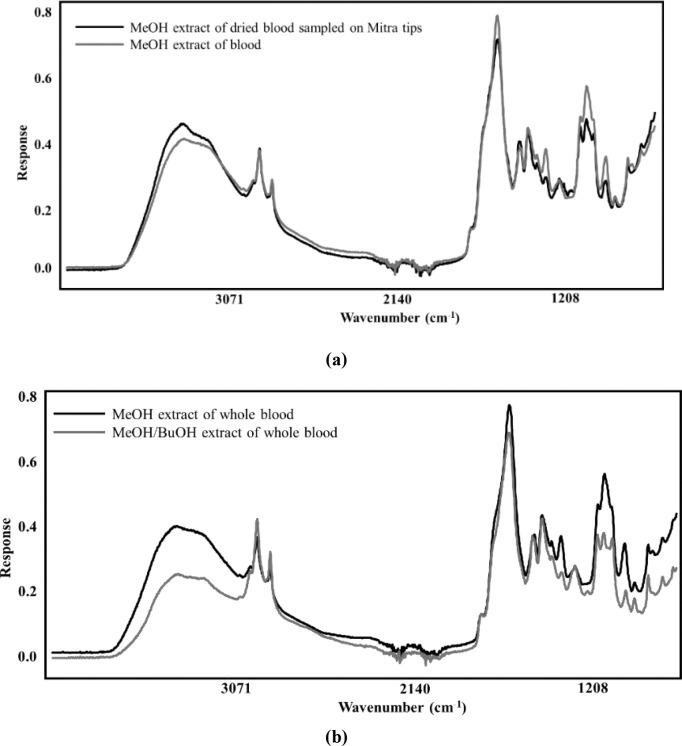
FTIR spectra of (a) MeOH extract from VAMS samples (black) and whole blood (gray), and (b) MeOH (black) and MeOH/BuOH extracts from whole blood (gray)

Metabolites from blood collected in additive-free blood collection tubes were extracted using MeOH precipitation in a similar way. The FTIR spectra of dried blood extracts from Mitra tips and liquid blood extracts appeared visually similar in both the spectral pattern and intensity (Figure 4a). Thus, the extraction recovery of metabolites from the substrate was good with minimal compromise in the IR response.

The MeOH spectra exhibits characteristic biomolecular features, including a broad band at 3600–3100 cm^−1^ corresponding to O-H stretching and amide-related vibration [13]. The region at 2800–3000 cm^−1^ reflects C-H stretching of methyl and methylene groups [37,38]. A distinct band at 1740 cm^−1^ is attributed to C=O stretching of lipid-related aldehydes and esters linkages [39,40], while bands at 1660–1580 cm^−1^ are associated with amide I and II vibrations and nucleic acid functional groups [9,39]. Within the fingerprint region (1500–700 cm^−1^), absorptions are mainly related to C–H bending (~1400 cm^−1^), phosphodiester vibrations (1245 and 1088 cm^−1^), and C–O/C–O–C stretching modes of lipids, polysaccharides, and nucleic acids near 1000 cm^−1^ [13].

As an alternative to MeOH/MTBE (Matyash) method, a single-phase MeOH/1-butanol (Alshehry) method was evaluated on whole blood to enhance extraction of lipids. While the Alshehry method enhanced nonpolar spectroscopic signatures, a few regions within the fingerprint region showed slightly lower intensities when compared to MeOH extract (Figure 4b). The performance of MeOH and MeOH/BuOH extraction solvents were compared, as discussed later in the Results section.

### 2.4 Classification analysis of FM vs HC using MeOH extracts from dried blood on VAMS substrate and whole blood

**Figure 5:**
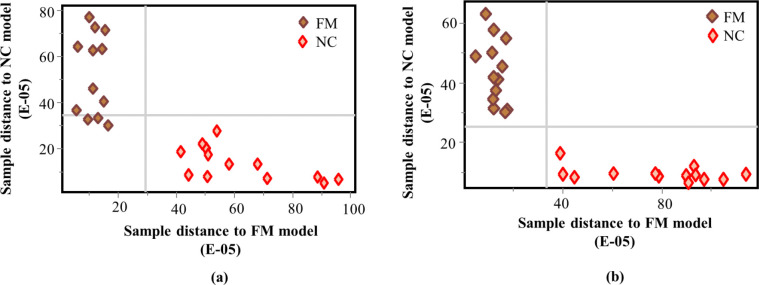
Cooman’s plot of 2-class SIMCA model obtained using MeOH extracts of dried blood on (a) VAMS substrate and (b) whole blood samples.

SIMCA, a statistical analysis for classification was used to visualize the differences in the spectral data of FM and HC extracts. Spectroscopic signatures in the 3000 – 2800 cm^−1^ and fingerprint regions (1750 to 750 cm^−1^) were used to generate the classification algorithms. The raw spectral data were mean-centered, normalized and SG second derivatized using 35 points. Figure 5a and Figure 5b shows the class distance or Cooman’s plot for the dried blood model and whole blood model respectively, visualizing the separation between FM and HC samples. Samples within each group clustered together and fell into their respective quadrants with no misclassifications. Furthermore, the interclass distance (ICD) for both models exceeded 3 (ICD of the dried blood model – 3.5 and ICD of the whole blood model – 4.1), indicating that the spectral data can be distinctly clustered into their corresponding classes.

**Figure 6:**
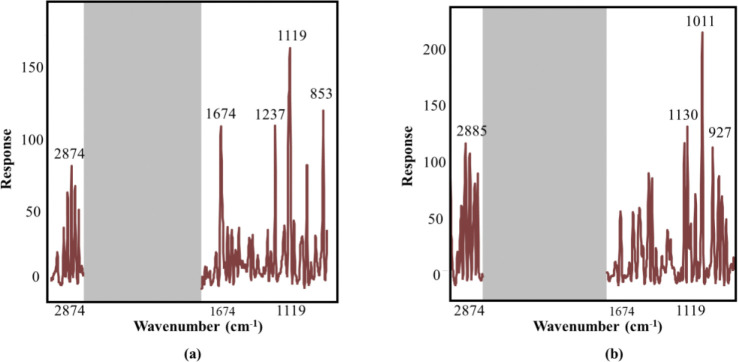
Discriminating power plot of 2-class SIMCA model differentiating FM and HC groups, obtained using MeOH extracts of dried blood on (a) VAMS substrate and (b) whole blood samples.

The discrimination power plot highlights the key features important in classifying samples. The wavenumbers 1119 cm^−1^, 853 cm^−1^, 1237 cm^−1^, 1674 cm^−1^ and 2874 cm^−1^ were responsible for interclass differences in MeOH extract from dried blood on VAMS tips (Figure 6a) while the wavenumbers 1011 cm^−1^, 1130 cm^−1^, 927 cm^−1^ and 2885 cm^−1^ were important in the model using whole blood MeOH extracts (Figure 6b). Five model factors were selected for both models, cumulatively explaining 97% to 98.5% of the total variation.

### 2.5 Spectroscopic analysis of a cohort of samples

A cohort of 40 FM, 20 RA and 10 HC were extracted using MeOH and MeOH/BuOH solvents and subsequently analyzed by FTIR. The MeOH/BuOH extracts were further analyzed by mass spectrometry. The results are discussed below.

#### 2.5.1 OPLS-DA classification of FM vs RA

**Figure 7:**
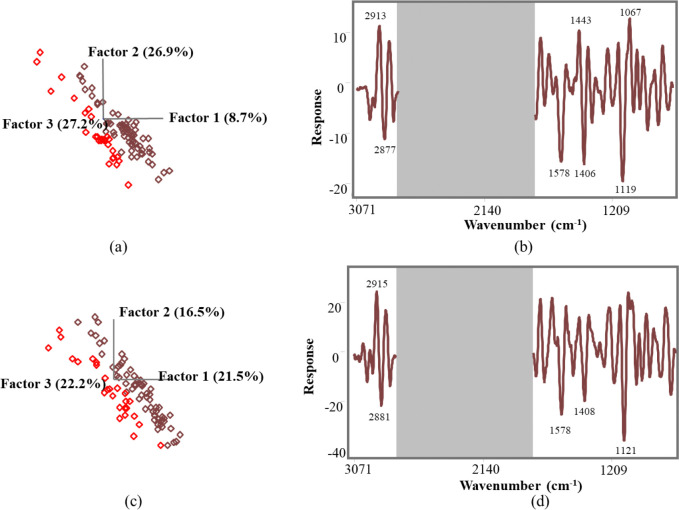
OPLS-DA scores plot with the first three factors obtained from FTIR spectral data of MeOH (a) and MeOH/BuOH extracts (c) demonstrating separation between FM (brown) and RA (red) samples. The corresponding OPLS-DA regression vectors highlight the key wavenumbers contributing to discrimination between FM and RA samples for MeOH (b) and MeOH/BuOH extracts (d).

The biphasic extraction method using MTBE/MeOH, as described by Matyash et al. produced a lipid enriched upper phase and higher lipid recoveries compared with the Folch method, however, it leached additives from plastic consumables which interfered with the FTIR results. Therefore, a one-phase extraction using MeOH/BuOH was applied to blood samples and evaluated by infrared analysis, in addition to MeOH extracts. As the total number of samples was higher for clinically similar syndromes, FM and RA, the classification algorithm OPLS-DA was used to model FM vs RA. The calibration OPLS-DA models produced distinct clusters for FM and RA samples for both the MeOH and MeOH/BuOH extraction methods (Figure 7a and 7c). The raw spectral data underwent similar pre-processing, including mean-centering, normalization and second derivative transformation (SG, 35 points).

Six model factors were selected that cumulatively described more than 84% of the spectral variance in both the MeOH and MeOH/BuOH models. The OPLS-DA classification method incorporates the regression capabilities of the PLS approach, where the response matrix is quantitative [13]. A leave-one-out cross validation approach was used to develop the calibration model in which each sample was sequentially excluded from the training set and predicted using a model built from the remaining samples. Eighty percent of the dataset was used to generate the calibration model, while the remaining 20% was used for external validation. External validation resulted in comparable classification accuracies of 83% for MeOH/BuOH extracts and 79% for MeOH extracts. The regression vector plots (Figure 7b and 7d) revealed similar wave numbers contributing to FM and RA discrimination in both models. Key discriminatory features were observed at 1119 cm^−1^, 1578 cm^−1^ (amide bands and amino acids), 1408 cm^−1^ (ν(COO^−^)), 2881 cm^−1^ and 2913 cm^−1^ (C-H stretching).

#### 2.5.2 SIMCA classification of FM/RA vs HC

**Figure 8:**
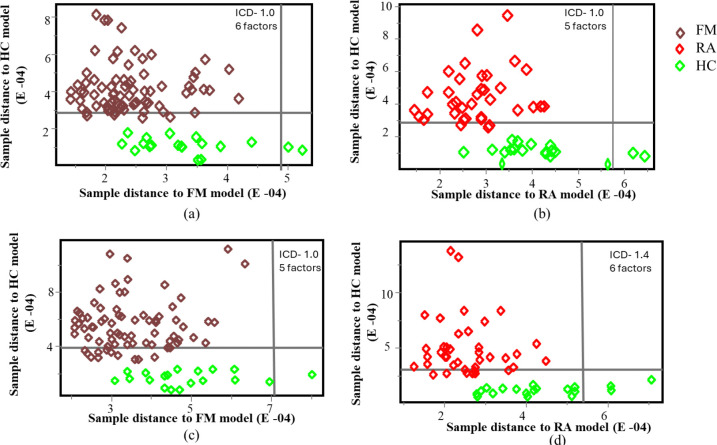
Cooman’s plot of the 2-class SIMCA models based on MeOH extracts: (a) FM vs HC and (b) RA vs HC; and MeOH/BuOH extracts: (d) FM vs HC and (e) RA vs HC.

As the number of samples in HC group was comparatively smaller, SIMCA was used to develop classification models of FM and RA versus HC. Figure 8 shows the class distance (Cooman’s plot) plot, illustrating the separation between FM and HC, and between RA and HC for the SIMCA models based on MeOH and MeOH/BuOH extracts of whole blood. Samples within each group clustered together, with ICD ranging from 1.0 to 1.4. The SIMCA classification models were built using 5 to 6 factors, cumulatively explaining more than 93% of the total spectral variance.

The corresponding discriminating power plots of the SIMCA models are shown in **Figure S2**. Wavenumbers 2985 cm^−1^, 1672 cm^−1^, 1451 cm^−1^ and 881 cm^−1^ were important for discriminating the groups in MeOH model **(Figure S2a and S2b)**, while wavenumbers 1667 cm^−1^, 1443 cm^−1^ and 1037 cm^−1^ were important for discriminating the groups in MeOH/BuOH model **(Figure S2c and S2d)**.

### 2.6 Mass spectrometry analysis

**Figure 9:**
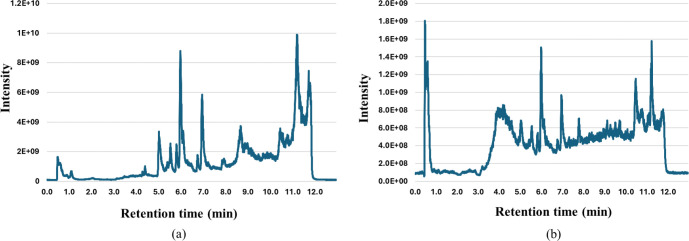
Representative total ion chromatogram obtained from (a) positive and (b) negative ionization mode in full scan MS analysis.

Untargeted LC–MS analysis resulted in complex chromatographic profiles, with metabolic features distributed throughout the full chromatographic run. Figure 9a shows the representative total ion chromatogram (TIC) obtained in positive ionization mode, while Figure 9b shows the representative TIC obtained in negative ionization mode from full-scan MS analysis of MeOH/BuOH extracts.

#### 2.6.1 Important features differentiating FM vs RA

Untargeted metabolomics analysis was conducted using LC-MS on MeOH/BuOH extracts of whole blood samples. After peak picking, retention time alignment and filtering, the analysis identified 294 and 570 compounds that were significantly different between the FM and RA groups in positive and negative mode metabolomics, respectively.

**Figure 10:**
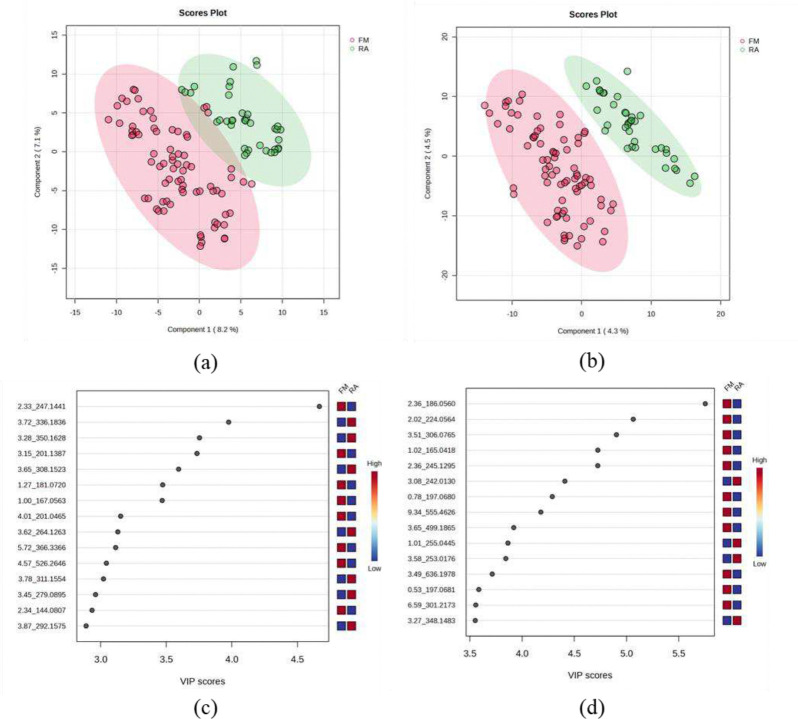
PLS-DA score plots of (a) positive-mode and (b) negative-mode metabolomics data showing differentiation between FM and RA groups. PLS-DA variable importance in projection (VIP) scores of (c) positive-mode and (d) negative-mode metabolomics data, highlighting the top 15 features important for differentiating the FM and RA groups.

A pattern recognition algorithm, PLS-DA was applied to reveal differences in metabolomic profiles between FM, RA and HC samples. The FM and RA groups were distinctly separated in the metabolomics scores plot (Figure 10a, positive mode metabolomics and Figure 10b, negative mode metabolomics). The variable importance in projection (VIP) score plots, Figure 10c and 10d show the top 15 metabolites contributing most to the classification in positive and negative mode, respectively. The legend of the plot indicates whether the compound is present at a relatively higher (red) or lower (blue) concentration in each group [41]. The top 60 features had VIP scores greater than 2.2. The top 20 features were further identified to determine their origin, whether they represent drugs or food metabolites accumulated due to chronic exposure, or metabolites endogenous to human blood. Table 3 lists the top 10 tentatively identified endogenous metabolites from the positive and negative mode comparative analysis among the FM and RA groups.

##### 2.6.1.1 Features in positive mode

Glucose metabolism and pentose phosphate pathway are markedly altered in RA, compared to FM, resulting in altered levels of hexoses (Compound ID 1.27_181.0720) and oxidation products of pentoses (ID 1.00_167.0563), particularly in RA [42–44]. N-acylethanolamines (NAEs) (ID 5.72_366.3366 and 6.18_342.3366), a class of signaling lipids that includes palmitoylethanolamide (PEA), anandamide (AEA) are known modulators of inflammation and pain and are altered in both RA and FM [45,46]. Additionally, SPB 21:1;2O (ID 6.18_342.3366), a member of the sphingoid base family, is part of a lipid class involved in immune regulation, and inflammatory signaling [47,48]. Supporting this, targeted metabolomics studies have identified sphingolipid-related metabolites linked to pain severity [49,50].

Inosine monosphosphate (ID 0.72_349.0543) is the central branch point of purine metabolism. Broad RA metabolomics reviews identify nucleotide/purine pathways as part of immune-cell metabolic reprogramming in inflamed synovium [42,51]. Targeted metabolomics links hypoxanthine (a direct IMP/inosine product) with pain and fatigue in FM, indicating altered purine handling [52].

Although cyclo(L-Phe-L-Val) is not explicitly annotated, phenylalanine-containing peptide derivatives fall within pathways that is shown to be dysregulated in RA [53]. Furthermore, dysregulated amino acid/BCAA metabolism and glutamine imbalance may result in altered levels of the tripeptide, Pro-Gln-Ile (ID 3.16_379.1936) and Glu-Val-Ala (ID 1.92_298.1408). Moreover, dysregulated indole metabolism in both conditions [52,54] is associated with altered levels of Indole-3-acrylic acid (ID 3.23_188.0706), a gut microbiota-derived tryptophan metabolite with known immunomodulatory properties.

##### 2.6.1.2 Features in negative mode

Glutathione (ID 3.51_306.0765) and L-Glutathione (reduced) (ID 1.81_306.0765) are altered in both RA and FM, reflecting disrupted redox balance linked to inflammation-driven oxidative stress [55] and mitochondrial stress [56]. Altered serotonin O-sulfate levels (ID 1.01_255.0445) were found in FM which reflects dysregulated serotonin turnover within the gut–brain axis, consistent with central sensitization and neurochemical imbalance underlying pain, fatigue, sleep disturbance, and mood symptoms [57]. Reduced levels of Eicosapentaenoic acid (ID 6.59_301.2173) in RA suggest increased utilization to promote anti-inflammatory action [58] while lower levels of 3-carboxy-L-tyrosine (ID 2.02_224.0564) and N-carbamoylhistidine (ID 0.78_197.0680) in RA is possibly due to inflammation-driven amino-acid modification, turnover and oxidative stress [59]. Furthermore, 7,8-Dihydro-2,4(1H,3H)-pteridinedione (ID 1.02_165.0418) was also found to be lower in RA, it is structurally related to the pteridine family which includes intermediates connected to oxidative stress and immune-cell redox regulation [60]. The elevated levels of S-[2-({N-[(2S)-2-Hydroxy-3,3-dimethyl-4-(phosphonooxy)butanoyl]-β-alanyl}amino)ethyl] (3R)-3-hydroxyoctanethioate (ID 3.65_499.1865) in FM may indicate altered fatty-acid metabolic flux [61] and mitochondrial inefficiency [62], leading to accumulation of phosphopantetheine-linked lipid intermediates.

##### 2.6.2 Important features differentiating FM/RA vs HC

After data cleanup, the analysis identified 443 and 711 compounds that were significantly different between the FM and HC groups in positive and negative mode metabolomics, respectively, while 516 and 897 compounds differed significantly between the RA and HC in positive and negative mode metabolomics, respectively.

**Figure 11:**
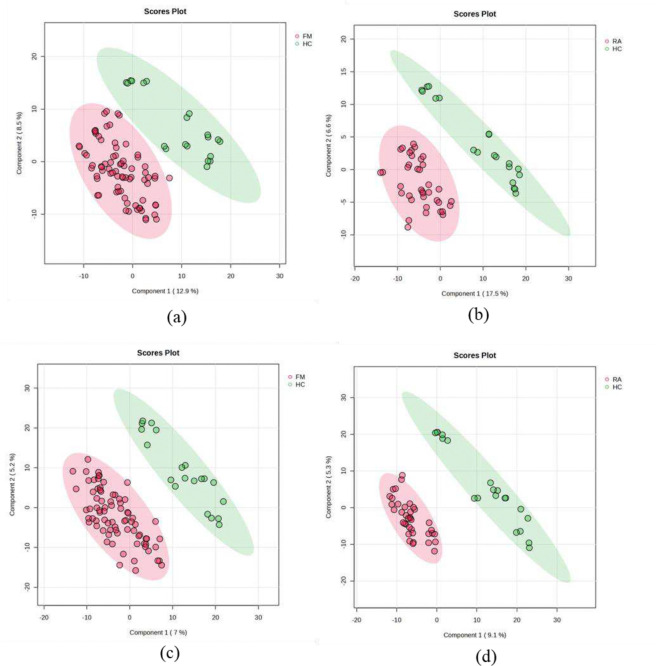
PLS-DA score plots of positive-mode metabolomics data showing differentiation between (a) FM vs HC and (b) RA vs HC groups and negative-mode metabolomics data showing differentiation between (c) FM vs HC and (d) RA vs HC groups.

The FM/RA and HC groups showed clear clustering in the metabolomics scores plot (Figure 11a and 11b, positive mode metabolomics and Figure 11c and 11d, negative mode metabolomics). The VIP score plots, **Figure S3** presents the top 15 metabolites contributing most to classification in positive and negative mode metabolomics. As in the previous section, these features were further identified to identify endogenous blood metabolites. Table 4 and 5 lists the top 10 tentatively identified endogenous metabolites from the positive and negative mode comparative analysis between the FM/RA and HC groups.

##### 2.6.2.1 Features in positive mode

Altered levels of N-Fructosyl phenylalanine (ID 1.11_328.1391) and N-Fructosyl isoleucine (ID 0.91_276.1442) which belong to fructosamine class, were observed. Fructosamines are stable ketoamines formed in human blood through the non-enzymatic glycation of circulating proteins and amino acids [63]. Cardiolipins (CL) (ID 3.39_809.3679) are central to mitochondrial membrane integrity and are highly sensitive to oxidation, changes in CL levels may indicate mitochondrial dysfunction and oxidative stress [64,65]. Altered levels of glutamate (ID 0.99_261.1446) were observed, consistent with metabolomic studies suggesting changes in neurotransmitter metabolism [66].

Studies have reported significantly altered lipid metabolism and increased oxidative stress in FM and RA compared with HC [67,68]. Recently, a dual role of lysophosphatidylserine (LPS) has been identified, it participates in the initiation of acute inflammation and also acts as a pro-resolving lipid mediator involved, facilitating the transition from an inflammatory to a restorative environment [69]. In the latter context, LPS produced by aging apoptopic neutrophils promotes their clearance by macrophages [69], supporting anti-inflammatory responses. In the present study, levels of LPS (ID 3.60_622.3480, ID 7.26_830.5503, ID 6.53_646.4079) and PE (ID 0.93_314.1710) were altered in FM and RA subjects compared to the control group. Altered amino acid metabolism in FM [70] may account for the observed changes in oligopeptide levels in FM vs HC (ID 3.74_326.2075, ID 3.22_372.1861 and ID 1.00_283.1290) and RA vs HC (ID 3.59_312.1918 and ID 3.65_217.1547). Acylcarnitines (ID 0.67_204.1230), including 3,4,5-Trihydroxypentanoylcarnitine (ID 0.91_276.1442 and ID 0.90_292.1401) have been observed in subjects with fatty acid metabolism disorders [71–74]. Carnitine plays an important role in energy production, and its deficiency may lead to energy deficits, resulting in fatigue and weakness [74].

##### 2.6.2.2 Features in negative mode

As shown in Table 4, the steroid metabolites testosterone sulfate (compound ID 3.85_367.1582) and androsterone sulfate (compound ID 4.07_369.1739) were both decreased in FM patients compared with HC, reflecting potential alterations in androgen metabolism consistent with prior reports of reduced androgen levels in women with FM and links between lower androgen status, pain severity, and HPA-axis dysregulation [75]. A cluster of di- and oligopeptides was also markedly depleted in FM, including Ser-Phe (ID 1.98_251.1037), Leu-Phe (ID 3.51_277.1558), the RGD-containing hexapeptide Gly-Arg-Gly-Asp-Ser-Pro (ID 3.92_586.2548), and a larger peptide (ID 3.59_238.0775), aligning with broader disturbances in amino-acid homeostasis and potentially influenced by altered gut microbiota, intestinal absorption, proteolytic activity, and barrier integrity [76–80]. Peptide-like feature containing motifs found in neuropeptide families (Compound ID 0.90_292.1401) was also altered, their structures resemble pyroglutamyl- and histidyl-prolinamide peptides involved in neuroendocrine regulation and central monoaminergic signaling [81]. Finally, two lysophosphatidic-acid species with compound ID 8.37_555.3424 and ID 8.86_597.3894 were altered, possibly due to oxidative-stress and lipid-peroxidation phenotypes in FM [82,83].

The MS/MS spectrum of the features listed in Table 6 showed fragment ions (≥ 3) that were consistent with reference spectra matched against the databases, supporting a putative identification at MSI level 2. These features primarily consisted of short chain peptides (dysregulated amino acid), inosine monosphosphate (purine metabolism), indole-3-acrylic acid (gut microbiota-derived tryptophan metabolite), acetyl-L-carnitine (fatty acid metabolism) and testosterone sulfate.

### 2.7 Correlating FTIR spectral data to important features from MS

PLSR statistical analysis was performed to build regression models, correlating the FTIR spectral data of MeOH/BuOH extracts from whole blood samples with the normalized areas of important MS features. This correlation of the top metabolites suggests that FTIR can capture molecular information associated with these compounds and is sensitive to the changes in their levels, thereby enabling their quantification. Table 7 lists the metabolites, their tentative IDs and the performance of regression models obtained using MeOH/BuOH extracts from whole blood samples.

**Figure 12:**
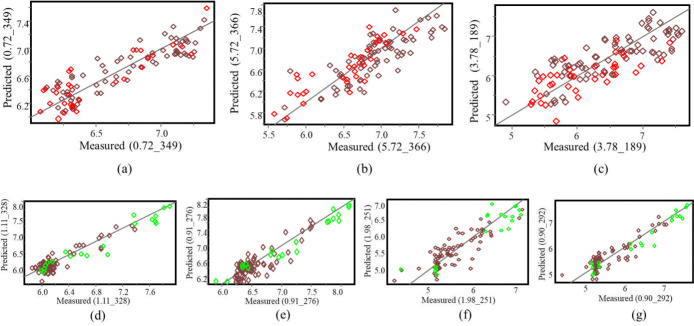
PLSR regression plots correlating the FTIR spectral data of MeOH/BuOH extracts from whole blood samples with the normalized areas of MS features as listed in Table 5

As seen in the correlation plots (Figure 12), the data points scattered well along the regression line in the PLSR models, indicating a good fit. Figure 12a – 12c represent regression plots of important MS features in the classification model comparing FM versus RA, while figure 12d – 12g represents regression plots of important features in the classification model of FM versus HC. The PLSR models exhibited good correlation of FTIR spectral data with the MS intensities, with correlation coefficient (R ≥ 0.79) and standard error of cross validation (SECV ≤ 0.30). A high correlation coefficient indicates improved predictive accuracy, while a low root mean square error represents better precision and predictive capability of the regression model [84].

## Discussion

While several studies have reported the application of vibrational spectroscopy for medical diagnostics and biomarker detection in biofluids (urine, serum, plasma, saliva), the experimental procedures typically use the liquid samples directly for rapid analysis [85–87]. However, these biofluids are complex aqueous solutions containing various components with a wide range of molecular weights. Such complexity can result in dominant signals that can mask those from small molecules of interest, making their identification difficult during statistical analysis. In this study, we evaluated different organic solvents for extracting metabolites from dried and whole blood samples and investigated their effects on FTIR spectral profiles. Subsequently, we developed an extraction protocol suitable for incorporating whole blood and VAMS technology into a typical untargeted, vibrational spectroscopy-based metabolomics workflow designed for global metabolome analysis. Furthermore, we correlated spectral data obtained from this high-throughput technique with multi-omics MS data to develop predictive regression models quantifying key markers. By integrating these multiple omics approaches, we address challenges associated with various confounding factors and provide a rapid, robust and reliable diagnostic methodology.

Understanding biological samples, from collection through processing and final analysis is crucial to ensure high-quality, accurate and reliable results and to minimize errors. Knowledge of analytical instruments being used is equally important, as this determines the required level of sample preparation, protocol compatibility and potential sources of analytical interference. For example, in MS analysis, signals from processing blanks during batch extraction can be subtracted from sample data. Although, for vibrational spectroscopy, particularly when developing predictive models, it is preferable that the spectral signals are authentic to the sample and free from external artifacts. Running process blanks on vibrational spectrometers can help identify potential contaminants and background noise. In the present study, possible factors that could interfere with the FTIR spectra included components from Whatman card (dried blood samples), EDTA from anticoagulant blood collection tubes and hemolytic particulates that may affect spectral consistency. To address these issues, blood was collected in additive-free tubes, sampled on novel VAMS substrate and extraction protocols modified to minimize hemolysis of RBCs.

Water induced hemolysis in blood samples, leading to inhomogeneous particulates that interfered with FTIR signals. Extraction using MeOH, a polar protic solvent, exhibited rich spectral signatures and high intensity across both the functional group (3700 – 2700 cm^−1^) and fingerprint region (1800 – 750 cm^−1^) compared with other solvents (1% FA, 1% NH_4_OH, EtOAc, Acetone and AcN). Various mixtures of MeOH with relatively non-polar solvents (AcN, EtOAc, MTBE and BuOH) were tested to improve the extraction of mid to non-polar metabolites. Extraction with MeOH/BuOH produced well-defined FTIR spectral profile across both the functional group and fingerprint region. In contrast, other solvent mixtures yielded well-defined nonpolar spectroscopic signatures (3000 – 2800 cm^−1^, C-H stretching vibrations, and 1730 cm^−1^, C=O stretching) but reduced intensities in the fingerprint region. MeOH/BuOH extracts provide slightly better classification, with an ICD of 4.6. Butanol, being less polar and having lower water solubility, likely extracted additional lipophilic compounds. The biphasic extraction method could not be used for FTIR analysis as it employed a higher concentration of MTBE to MeOH (3:1), which led to the leaching of extractables from plastic labware and interference with FTIR signals.

Blood represents a complex biological matrix, in addition to important markers, it contains metabolites of drugs and hormones that vary according to age and sex [88]. FTIR captures signals from the diverse array of molecules, while mass spectrometry resolves chemical mixtures for detailed metabolomic analysis of such highly complex samples. Dipeptides, indole 3 acrylic acid, glutathione and glucose were among the top features identified by MS, differentiating the clinically similar, FM and RA syndromes. By integrating MS data with IR spectra and developing regression models to predict levels of key metabolites, the algorithm can be further guided to focus on variance associated with key markers. This approach may ensure that the model captures variance related to metabolites relevant to the syndrome, while minimizing the influence of extraneous factors such as age, sex and medications use. Pathway-level inspection suggested perturbations in oxidative stress, inflammation modulators, as well as purine, amino acid and free fatty acid metabolism, consistent with prior metabolomic studies in fibromyalgia. The levels of these metabolites were correlated with FTIR spectral data using a PLSR algorithm. The resulting regression models exhibited correlation coefficient (R) greater than 0.70 and a low standard error of cross validation (SECV ≤ 0.30), indicating that FTIR effectively captures variance associated with key markers and can serve as a rapid method for estimating their levels as determined by MS. Thus, in addition to developing classification algorithm, generating predictive regression models for key metabolites can provide a rapid, robust and reliable diagnostic workflow. Overall, our results demonstrate the ability of vibrational spectroscopy and mass spectrometry to identify unique metabolites and facilitate point-of-care diagnostics solution for FM.

The limitations of this study include its small cohort size. Incorporating a larger number of samples that represent diverse clinical populations will provide more reliable, real-world performance metrics. External validation of the trained algorithm is also necessary to further assess its robustness and reliability. Additionally, the features identified by MS are putative and based on database matching, they must be further verified to define them as definitive markers.

## Materials and methods

### Subject enrollment

Participants were drawn from an ongoing multi-institutional biomarker discovery program investigating metabolic signatures of chronic pain and rheumatic disease conducted at The University of Texas at Austin and The Ohio State University. The program enrolls patients from rheumatology clinics as well as community volunteers serving as healthy controls. Diagnoses of fibromyalgia are established by treating rheumatologists using 2016 American College of Rheumatology criteria. Healthy controls were screened to exclude chronic rheumatic or pain conditions and furthermore were determined to be free of any concurrent prescribed pharmaceuticals. All participants provided informed consent under an Institutional Review Board (IRB)–approved protocol. The study was approved by the University of Texas at Austin Institutional Review Board (IRB #2020030008) and conducted in accordance with the Declaration of Helsinki. All participants provided informed consent.

Self-reported symptoms were collected from all participants using the Revised Fibromyalgia Impact Questionnaire (FIQR), a 10-item self-report measure that assesses physical function, work ability, depression, anxiety, sleep quality, pain, stiffness, fatigue, and overall well-being [89]. Depressive symptoms were evaluated using the Beck Depression Inventory (BDI), a 21-item questionnaire designed to measure the psychological and behavioral aspects of depression [90]. The Symptom Impact Questionnaire-Revised (SIQR), an FM-neutral adaptation of the FIQR, was also used and does not presume a diagnosis of fibromyalgia [91]. The Central Sensitization Inventory (CSI) is a two-part patient-reported outcome measure that assesses somatic and emotional symptoms [92]. These values are reported in Table 8.

### Sample collection

Blood samples were collected intravenously in additive free tubes (BD vacutainer ^®^, Nebraska, USA). To ensure accurate and consistent sampling volume, a 30 μl aliquot of blood was pipetted onto parafilm and wicked onto a Neoteryx Mitra device (Neoteryx, CA, USA) (30 μL total collection volume tip) employing VAMS technology, according to the manufacturer’s guidelines [93]. Whole blood samples and dried blood samples on the VAMS substrate were shipped to the Rodriguez-Saona Vibrational Spectroscopy laboratory at The Ohio State University and stored at −80 °C until extraction.

### Chemicals

All materials were obtained from Fisher Scientific (Thermo Fisher Scientific,New Jersey, USA). Nanopure water was obtained using a Milli-Q water purification system. All chemicals and solvents were of analytical grade or higher purity.

### Metabolite extraction from VAMS substrate

Seven different solvents were selected to disrupt the ionic and hydrophobic interaction between the analytes and the VAMS substrate and subsequently evaluate the extraction protocol. These solvents included water, 1% Formic Acid (FA) and 1% Ammonium Hydroxide (NH_4_OH) to reduce ionic interactions, ethyl acetate (EtOAc) to help break Van der waals forces and water miscible organic solvents such as acetone, acetonitrile (AcN) and methanol (MeOH) for protein precipitation [16,17].

Each VAMS tip was placed into microcentrifuge (Eppendorf) tube. Metabolites were extracted by adding 300 μl of the respective ice-cold extraction solution to the VAMS substrate. Following solvent addition, samples were vortexed for 15 seconds and sonicated in an ice-bath for 15 minutes. The VAMS tips were then transferred to new Eppendorf tube for re-extraction. An additional 300 μl of extraction solvent was added, and the samples were sonicated for 10 mins in an ice-bath. Finally, the VAMS tips were removed, and the combined extracts were vacuum dried for 3 hrs to obtain a pellet.

### Metabolite extraction from whole blood

Additive-free tubes containing whole blood were centrifuged at 4200 rpm for 10 min at room temperature. Metabolites were extracted by mixing 30 μl of the supernatant with ice-cold solvent which constituted 300 μl MeOH for MeOH extraction and for the Alshehry method, sequential addition of 300 μl of each MeOH and 1-Butanol (MeOH/BuOH). Samples were vortexed for 15 sec and sonicated in an ice bath for 15 min, followed by centrifugation at 16000 g for 10 min at 4°C. The clear supernatant was carefully transferred into another microcentrifuge tubes, taking care to avoid hemolytic particulates. The extracts were then dried to obtain a pellet.

The Matyash extraction method was used for biphasic extraction protocol. Briefly,150 μl of ice-cold MeOH was added to 30 μl of supernatant, followed by the addition of 500 μl of Methyl tert-butyl ether (MTBE). Samples were vortexed (15 sec) and sonicated in an ice bath for 10 min, followed by addition of 150 μl nanopure water (18.2 MΩ). Samples were vortexed, sonicated and centrifuged (16000 g for 5 min at 4°C). The resulting polar and non-polar fractions were carefully aliquoted into clean Eppendorf tubes and vacuum dried.

### Infrared Spectroscopy Analysis

FTIR measurements were performed with a portable Agilent 4500 Series FTIR spectrometer (Agilent Technologies, Santa Clara, CA, USA) with a triple-bounce diamond ATR module, covering 4000–700 cm^−1^. The sampling area was a 2 mm diameter surface with a 200 μm active region and an estimated penetration depth of ~6 μm at 17 cm^−1^. The system used a zinc selenide beam splitter, a high-throughput Michelson interferometer, and a thermoelectrically cooled dTGS detector [8]. Prior to analysis, the dried pellets were reconstituted with 3 μl of MeOH and 3 ul of water. For the MeOH/BuOH extracts, reconstitution was performed using 4ul of a 1:1:8 H_2_O: Toluene: MeOH mixture and 2ul of water. Samples were vortexed for 15 sec and sonicated for 30 sec. A 2 μL aliquot of the resulting suspension was then placed onto the ATR crystal and vacuum-dried to form a thin film. The crystal was wiped with 70% ethanol and a background spectrum collected before each run. Spectra were recorded at 4 cm^−1^ resolution with 64 co-added scans to improve signal-to-noise. Spectral acquisition were randomized to minimize batch effects.

### Untargeted LC-MS/MS metabolomics and lipidomics analysis

Untargeted LC-MS/MS metabolomics was performed on a Vanquish UHPLC coupled to an Orbitrap Exploris 480 mass spectrometer (Thermo Fisher Scientific, MA, USA), equipped with high-flow heated electrospray ionization (HESI) probes. An Accucore C18 2.1 × 100 mm, 2.6 μm column (Thermo Fisher Scientific, USA) was used for the analysis. Mobile phase A consisted of 0.1% formic acid in nanopure water, while mobile phase B consisted of 0.1% formic acid and 10 mM ammonium formate in 30/70 IPA/acetonitrile. Solvent B was modified from the standard metabolomics solvent to enable separation of relatively nonpolar compounds extracted using MeOH/BuOH solvent. The binary mobile phase system were used with the gradient as follows- 0–1 min, held at 2% B; 1–2 min, 2% to 5% B; 2–3 min, 5% to 50% B; 3–4 min, 50% to 65% B; 4–8 min 65% to 90%; 8–11 min, held at 90% B; 11–11.1 min 90% to 2%, 11.1–13 min, held at 2% B. The flow rate was 0.4 mL/min. The column temperature was set at 45 °C. An injection volume of 5 μL was used for LC-MS/MS analysis. All the samples were analyzed in a random order. The quality control sample was injected at the beginning of the sequence and after every 15 sample injections. Each sample was analyzed in duplicates.

The HESI positive mode and negative data were collected separately. H-ESI spray voltage was set at 3500 V for positive ion mode and 2500 V for negative ion mode. The mass spectrometer settings were as follows: Sheath gas, auxiliary gas, and sweep gas were set at 35, 7, and 1 arbitrary units (Arb), respectively. Ion transfer tube at 320°C; vaporizer temperature at 275 °C. The full scan MS spectra in the range of 100–1500 m/z with 50% RF lens and 60,000 mass resolution were collected for each sample. The AcquireX program with DDA mode was used to collect MS/MS spectra from QC samples. Specifically, a standard AGC, a normalized HCD collision energy of 30%, 50%, and 80%, a resolution of 60k, and a mass isolation width of 1.5 m/z were used. The top 10 MS/MS scans were recorded from signals above the threshold of 100,000 ion counts.

### Multivariate data analysis of MS and FTIR

Compound Discoverer (v 3.4m Thermo Fisher Scientific, USA) was used to perform data processing of MS, including peak picking, retention time alignment, background removal, ion extraction and integration, and putative identity annotation. In addition, the chemical features (labelled as RT_m/z) with ion intensity below 10,000 and RSD higher than 30% in QC were removed. Both accurate mass and MS/MS fragment ions were used to match metabolites on LC/MS database, including MZCloud, HMDB and ChemSpider. The mass accuracy of 10 ppm for precursor ions and 15 ppm for product ions was used to annotate compounds. Classification analysis of the pre-processed MS features was performed using Partial Least-Squares Discriminant Analysis (PLS-DA) in the open-source MetaboAnalyst 6.0 [94].

FTIR spectral analysis was performed using multivariate statistical software (Pirouette^®^ version 5.0, Infometrix Inc., Woodville, WA, USA). Soft independent modelling of class analogy (SIMCA) was applied for supervised classification, with FM and HC samples assigned to class 1 and class 2, respectively. The discriminating power plot generated by SIMCA highlighted the key infrared wavenumbers most relevant for distinguishing between the sample classes [95]. Cooman’s plot was used to visualize the clustering of the two classes and the interclass distance [95].

Partial Least Squares Regression (PLSR) was used to correlate the normalized areas of the target metabolites (dependent variables) with the FTIR spectral data (independent variables). PLSR was selected due to its capacity to handle collinear and high-dimensional spectral predictors, as it transforms the original spectral matrix X (independent variables) into a reduced set of orthogonal latent variables that represent systematic spectral variation while preserving its predictive relationship with the response matrix Y (dependent variables) [96]. A log-linear regression model was developed in which the dependent variables were log10-transformed. Logarithmic transformation not only transforms variables into approximately normal distribution but also linearizes the relationship between X and Y while preserving the underlying non-linear structure [97]. PLSR model performance was evaluated using correlation coefficient (R) and standard error of cross-validation (SECV). The correlation coefficient (R) quantifies the agreement between the predicted and reference MS areas, with values closer to 1 indicating a stronger linear relationship [98]. SECV represents the average deviation between predicted and measured values during internal validation, where lower values indicate greater predictive accuracy [99].

Prior to SIMCA and PLSR analysis, FTIR spectral data were preprocessed to improve signal quality and model robustness [84]. Pre-processing included normalization, mean-centering and second derivative transformation using the Savitzky-Golay (SG) polynomial fitting algorithm, with a 35-point window for SIMCA model and a 25-point window for PLSR model, to enhance spectral resolution and reduce baseline effects [100]. Statistical models were internally validated via leave-one-out cross-validation.

## Supplementary Material

This is a list of supplementary files associated with this preprint. Click to download.

• SupplementaryFiguresv2.docx

## Figures and Tables

**Table 1:** Observations regarding the FTIR spectral profiles of extracts obtained using various aqueous and organic-based solvents

Solvent	Comment
1% FA, 1 % NH4OH	Brownish-red extract, not suitable for spectroscopic analysis and requires further purification
AcN, Acetone, EtOAc	Signals in the hydrophobic region, weak fingerprint signals
MeOH	Good signals in the hydrophobic and fingerprint region. A clear extract enables spectroscopic and MS analysis without the need of further purification.
Water	Reddish extract, requires further purification. Extracts macromolecules with dominant signals from LMFs and the hydrophobic region that are not well defined.

**Table 2:** FTIR spectra of various solvent mixtures (gray) compared with the FTIR spectra of pure MeOH extract (black), along with the corresponding key observations. As the polarity of the extractant is reduced, the nonpolar spectroscopic signatures, primarily the functional group region (3000 – 2800 cm^−1^, C-H stretching vibrations) and 1730 cm^−1^ (C=O stretching) become more defined, while the fingerprint spectral signatures decrease in intensity.

Solvent mixtures	FTIR spectra of the solvent mixture extract and pure MeOH extract	Observation
90% MeOH	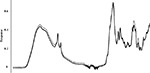	Spectral pattern and intensity are similar to the pure MeOH extract
70:20:10 MeOH:MTBE:H2O	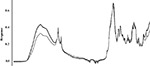	Spectral pattern and intensity are similar to the pure MeOH extract, with better definition in 3000 – 2800 cm^−1^, C-H stretching vibrations.
80:20 MeOH:MTBE	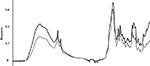	Well-defined nonpolar spectroscopic signatures (3000 – 2800 cm^−1^, C-H stretching vibrations, and 1730 cm^−1^, C=O stretching) but lower intensities in fingerprint region
70:30 MeOH : AcN	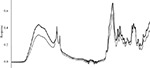	
70:30 MeOH:MTBE	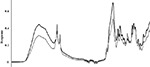	
50:50 MeOH : EtOAc	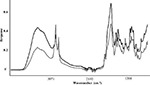	

**Table 3:** Top 10 tentatively identified endogenous blood metabolites with the highest VIP scores differentiating between FM and RA syndromes.

Positive FM vs RA		Negative FM vs RA	
Feature (RT_m/z)	Tentative ID	Feature (RT_m/z)	Tentative ID
2.33_247.1441	Cyclo(L-Phe-L-Val)	3.51_306.0765	Glutathione
1.27_181.0720	D-Glucose, D-Mannose, myoInositol, Fructose-1-Phosphate	1.01_255.0445	Serotonin O-sulfate
1.00_167.0563	Ribonic acid, Xylonic acid, Arabinonic acid	6.59_301.2173	Eicosapentaenoic acid
5.72_366.3366	NAE 21:2	1.81_306.0765	L-Glutathione (reduced)
6.18_342.3366	NAE 19:0, SPB 21:1;2O	1.92_298.1408	Glu-Val-Ala
0.72_349.0543	Inosine monosphosphate	2.02_224.0564	3-Carboxy-L-tyrosine
3.16_379.1936	Pro-Gln-Ile	1.02_165.0418	7,8-Dihydro-2,4(1H,3H)-pteridinedione
3.23_188.0706	Indole-3-acrylic acid	0.78_197.0680	N-Carbamoylhistidine
		3.65_499.1865	S-[2-({N-[(2S)-2-Hydroxy-3,3-dimethyl-4-(phosphonooxy)butanoyl]-β-alanyl}amino)ethyl] (3R)-3-hydroxyoctanethioate

**Table 4:** Top 10 tentatively identified endogenous blood metabolites with the highest VIP scores differentiating between FM and HC syndromes.

Positive FM vs HC		Negative FM vs HC	
Feature (RT_m/z)	Tentative ID	Feature (RT_m/z)	Tentative ID
3.85_271.2057	Androsta-4,16-dien-3-one	3.85_367.1582	Testosterone sulfate
1.11_328.1391	N-Fructosyl phenylalanine	4.07_369.1739	Androsterone sulfate
0.91_276.1442	N-Fructosyl isoleucine	1.98_251.1037	Ser-Phe
0.67_204.1230	Acetyl-L-carnitine	0.90_292.1401	N-Acetylhistidylprolinamide
3.39_809.3679	CL(26:3), CL(28:6)	3.92_586.2548	Gly-Arg-Gly-Asp-Ser-Pro
0.99_261.1446	L-γ-Glutamate / γ-Glutamate, γ-Glutamyl dipeptides (major endogenous class)	3.41_807.3529	PIP(O-28:6), PIP(P-28:5), Thr-Phe-Glu-Ala-Gly-Asp-Gly-Ile
3.74_326.2075	SPHP(d14:0), Isoleucyl-prolyl-proline, L-Proline	3.59_238.0775	Thr-Phe-Glu-Ala-Gly-Asp-Gly-Ile
3.60_622.3480	PC(23:4), PE(26:4), LPS(P-27:7)	8.37_555.3424	LPA(27:4), PA(O-27:4), PA(P-27:3)
3.22_372.1861	Pyroglutamyl-glutamyl-proline amide	8.86_597.3894	LPA(30:4), PA(O-30:4), PA(P-30:3)
1.00_283.1290	Tyr-Thr	3.51_277.1558	Leucylphenylalanine

**Table 5:** Top 10 tentatively identified endogenous blood metabolites with the highest VIP scores differentiating between RA and HC syndromes.

Positive RA vs HC		Negative RA vs HC	
Feature (RT_m/z)	Tentative ID	Feature (RT_m/z)	Tentative ID
3.59_312.1918	Val-pro-pro, Phe-Lys	1.98_251.1037	Ser-Phe
7.26_830.5503	PS 18:0_18:0	3.85_367.1582	Testosterone sulfate
0.91_276.1442	3,4,5-Trihydroxypentanoylcarnitine	2.36_186.0560	Indole-3-acrylic acid
5.72_366.3366	NAE 21:2	3.33_261.1245	Phe-Pro
0.93_314.1710	LPE O-7:0	3.85_757.3058	5-Oxo-L-prolyl-3-(4H-imidazol-4-yl)-L-alanyl-L-tryptophyl-L-seryl-L-tyrosylglycinamide
3.85_271.2057	Androsta-4,16-dien-3-one	2.02_224.0564	3-Carboxytyrosine
6.53_646.4079	PS O-16:3_11:0	0.90_292.1401	3,4,5-Trihydroxypentanoylcarnitine
3.65_217.1547	Valylvaline	3.41_807.3529	L-Threonyl-L-phenylalanyl-L-α-glutamyl-L-alanylglycyl-L-α-aspartylglycyl-L-isoleucine
8.29_401.3414	7-Ketocholesterol	3.38_571.7419	hexacosanoyl-CoA
0.67_204.1230	Acetyl-L-carnitine	3.44_589.2625	LPI 14:0

**Table 6:** List of features with fragment ions (atleast 3) matching reference spectra in mzCloud library.

Model	Feature ID	Name
Positive FM-RA	0.72_349.0543	Inosine monosphosphate
	3.16_379.1936	Pro-Gln-Ile
	3.23_188.0706	Indole-3-acrylic acid
Positive FM HC	0.67_204.1230	Acetyl-L-carnitine
Negative FM RA	1.81_306.0765	L-Glutathione (reduced)
	1.92_298.1408	Glu-Val-Ala
Negative FM HC	3.85_367.1582	Testosterone sulfate
	1.98_251.1037	Ser-Phe
Negative RA HC	3.85_367.1582	Testosterone sulfate
	2.36_186.0560	Indole-3-acrylic acid
	3.33_261.1245	Phe-Pro

**Table 7:** Tentative identification of metabolites and the performance of PLSR models obtained using MeOH/BuOH extracts from whole blood samples

Model	Feature ID	Tentative metabolite	N	Rcv	SECV
**Positive FM - RA**	**0.72_349**	Inosine monosphosphate	8	0.88	0.18
	**5.72_366**	NAE 21:2	9	0.84	0.27
**Negative FM - RA**	**3.78_189**	MAG 6:0	6	0.79	0.41
**Positive FM - HC**	**1.11_328**	N-Fructosyl phenylalanine	9	0.95	0.16
	**0.91_276**	N-Fructosyl isoleucine	8	0.93	0.19
**Negative FM - HC**	**1.98_251**	Ser-Phe	6	0.88	0.30
	**0.90_292**	N-Acetylhistidylprolinamide	5	0.93	0.26

**Table 8:** Clinical characteristics of all subjects.

	Age	N	BMI	BDI	CSI	FIQR	SIQR
**FM**	44.1 ± 14.6	40	30.3 ± 8.5	17.1 ± 11.0	58.8 ± 19.5	50.1 ± 20.5	36.3 ± 28.4
**RA**	45.4 ± 12.9	20	29.1 ± 6.1	9.3 ± 11.2	25.3 ± 23.0		37.2 ± 24.7
**HC**	25.3 ± 3.08	10	22.5 ± 2.3	1.0 ± 1.7	7.3 ± 8.1		1.7 ± 3.7

Values expressed as mean ± sd; N = number of subjects, Age (range). FM: fibromyalgia, RA: rheumatoid arthritis, HC: Healthy Control. BMI: body mass index. BDI: Beck depression index. CSI: Central Sensitization Inventory. FIQR: fibromyalgia impact questionnaire revised. SIQR: symptom impact questionnaire revised.

## Data Availability

The datasets generated during the study are available on request.
